# ERCC1/XPF Protects Short Telomeres from Homologous Recombination in *Arabidopsis thaliana*

**DOI:** 10.1371/journal.pgen.1000380

**Published:** 2009-02-13

**Authors:** Jean-Baptiste Vannier, Annie Depeiges, Charles White, Maria Eugenia Gallego

**Affiliations:** Génétique, Reproduction et Développement, UMR CNRS 6247, Clermont Université, INSERM U931, Aubière, France; Institut Jean-Pierre Bourgin, INRA de Versailles, France

## Abstract

Many repair and recombination proteins play essential roles in telomere function and chromosome stability, notwithstanding the role of telomeres in “hiding” chromosome ends from DNA repair and recombination. Among these are XPF and ERCC1, which form a structure-specific endonuclease known for its essential role in nucleotide excision repair and is the subject of considerable interest in studies of recombination. In contrast to observations in mammalian cells, we observe no enhancement of chromosomal instability in Arabidopsis plants mutated for either *XPF* (*AtRAD1*) or *ERCC1* (*AtERCC1*) orthologs, which develop normally and show wild-type telomere length. However, in the absence of telomerase, mutation of either of these two genes induces a significantly earlier onset of chromosomal instability. This early appearance of telomere instability is not due to a general acceleration of telomeric repeat loss, but is associated with the presence of dicentric chromosome bridges and cytologically visible extrachromosomal DNA fragments in mitotic anaphase. Such extrachromosomal fragments are not observed in later-generation single-telomerase mutant plants presenting similar frequencies of anaphase bridges. Extensive FISH analyses show that these DNAs are broken chromosomes and correspond to two specific chromosome arms. Analysis of the Arabidopsis genome sequence identified two extensive blocks of degenerate telomeric repeats, which lie at the bases of these two arms. Our data thus indicate a protective role of ERCC1/XPF against 3′ G-strand overhang invasion of interstitial telomeric repeats. The fact that the *Atercc1* (and *Atrad1*) mutants dramatically potentiate levels of chromosome instability in *Attert* mutants, and the absence of such events in the presence of telomerase, have important implications for models of the roles of recombination at telomeres and is a striking illustration of the impact of genome structure on the outcomes of equivalent recombination processes in different organisms.

## Author Summary

Telomeres are the specialised nucleoprotein structures evolved to avoid progressive replicative shortening and recombinational instability of the ends of linear chromosomes. Notwithstanding this role of telomeres in “hiding” chromosome ends from DNA repair and recombination, many repair and recombination proteins play essential roles in telomere function and chromosome stability. Among these are XPF and ERCC1, which form a structure-specific endonuclease known for its essential role in nucleotide excision repair and that is the subject of considerable interest in studies of recombination. In this study, we analyse the roles of the XPF/ERCC1 in telomere function and chromosome stability in the plant *Arabidopsis thaliana*, which, with its remarkable tolerance to genomic instability and sequenced genome, is an excellent higher eukaryotic model for these studies. Surprisingly, and in striking contrast to observations in mammalian cells, we observe no enhancement of chromosomal instability in Arabidopsis plants lacking either of these two proteins, which develop normally and show wild-type telomere length. However, *Atercc1* (and *Atrad1*) mutants profoundly affect the recombination of de-protected telomeres, dramatically potentiating chromosome instability. These results provide a striking illustration of the different outcomes and genomic impacts of the same recombination processes in different organisms.

## Introduction

Telomeres are the specific chromatin structures present at the ends of linear chromosomes [1]. They are known to play two main roles in the preservation of chromosomal integrity: avoiding terminal DNA sequence loss after replication and assuring that the chromosome ends are not recognized by the cellular machinery as DNA double-strand breaks [2]–[8]. In general, eukaryotic telomeres are composed of tandem repeats of a short sequence rich in G/C that terminates in a single strand 3′ overhang which can fold back and invade the duplex repeats to form the so called T-loop. A specific telomeric protein complex known as shelterin is implicated in the stabilization of the T-loop [9],^10^. In mammalian cells this complex includes the specific telomeric-DNA-binding proteins TRF1 and TRF2, which interact directly with duplex telomeric DNA, and POT1 which associates with the 3′ single stranded DNA. In most organisms telomeres are maintained by telomerase, a reverse transcriptase with a RNA subunit that serves as template for telomeric repeat synthesis. In the absence of telomerase, telomeres shorten with successive cell divisions, become non-functional and identified by the cell as damaged DNA, ultimately leading to genetic instability and cell death [11],[12].

In recent years, many other proteins known for a more general role in cellular metabolism have been found to associate to telomeres, notably proteins involved in DNA repair and recombination. These include MRE11/RAD50/NBS1, KU70/KU80, DNAPKcs, BLM/WRN and ERCC1/XPF and have been found associated with telomeres and to play important roles in telomere protection and/or homeostasis (reviews [5],[7],[13],[14]). In the work presented here our interest has focussed particularly on the ERCC1/XPF heterodimer, which has been shown to associate to telomeres through interaction with TRF2 protein in mammalian cells [15]. ERCC1/XPF is a structure-specific endonuclease, initially identified for its essential role in nucleotide excision repair (NER) in budding yeast [16]. ERCC1 and XPF are highly conserved proteins and, in addition to yeast (Rad1/Rad10), orthologs have been identified in many organisms including Arabidopsis (AtERCC1/AtRAD1) [17]–[22], *S. pombe* (Rad16/Swi10) [23],[24] and Drosophila (DmERCC1/MEI-9) [25],[26]. The ERCC1/XPF endonuclease activity specifically recognises double- to single-strand transitions in DNA, incising the 5′–3′ single-strand just after the junction (reviews by [27]–[30]). This DNA structure is a common element of homologous recombination intermediates and the 3′-ended G-strand overhang at telomeres is also a DNA structure of this type, although it is protected by the T-loop structure. In agreement with this, it has been shown that TRF2 is essential for T-loop stabilization, and its absence results in ERCC1/XPF -dependent, telomeric 3′ overhang loss [15].

Telomeres in most plant species are constituted of the repeat sequence TTTAGGG, initially identified in *Arabidopsis thaliana* [31]. Described plant telomeres vary in length from 2–9 Kb in Arabidopsis to 150 Kb in tobacco. The presence of G-overhangs has been detected in Arabidopsis and *S. latifolia* [32] and T-loops have been observed at telomeres of the garden pea, *Pisum sativum* [33]. Thus end-capping mechanisms seem to be conserved between mammals and plants. However, relatively little is known about plant telomeric proteins and in particular, the constituents of the plant shelterin complex have not been functionally identified [34]. Notwithstanding, a number of factors known for their roles in DNA repair such as the RAD50/MRE11 complex and the KU70/KU80 heterodimer has been found to play essential roles in protection of Arabidopsis chromosome ends [13],[14]. Given the conserved functional roles of the mammalian ERCC1/XPF proteins and the plant orthologs AtERCC1/AtRAD1 in DNA repair and recombination, we present here an analysis of the roles of AtERCC1/AtRAD1 in telomere homeostasis and chromosomal stability in Arabidopsis plants.

We demonstrate an essential role for the AtERCC1/AtRAD1 nuclease in the protection of shortened telomeres in *Attert* mutant plants. In striking contrast to XPF−/− and ERCC1−/− mammalian cells, Arabidopsis plants mutated for the *AtERCC1* or *AtRAD1* genes are viable and do not show any obvious defects in growth or development after more than 5 successive mutant generations. In the absence of telomerase, mutation of either *AtERCC1* or *AtRAD1* induces much earlier onset of developmental defects, correlated with increased genome instability. FISH analyses of mitotic anaphase figures shows that only 53% of the anaphase bridges in double mutant plants result from end-to-end chromosome fusions, compared to 91% in later generation *Attert* mutants with the same level of instability. Furthermore, 90% of the non end-to-end chromosome bridges are accompanied by large acentric DNA fragments in the double mutants. This simultaneous formation of a dicentric and an acentric chromosome is a consequence of recombination between telomeres and large interstitial blocks of degenerate telomeric sequences present on the right arms of chromosomes 1 and 4. We conclude that the endonuclease AtERCC1/AtRAD1 protects short telomeres from “destructive” homologous recombination in Arabidopsis plants.

## Results

### Absence of AtERCC1/AtRAD1 Accelerates Genomic Instability in Telomerase-Minus Arabidopsis Plants

Absence of TRF2 protein in mammalian cells leads to telomere uncapping and chromosome fusions. Such fusions require the presence of the ERCC1/XPF nuclease, which by eliminating the single-stranded 3′ G-strand overhang, generates the non-homologous end-joining (NHEJ) substrate [15]. We decided to check whether the AtERCC1/AtRAD1 proteins are required for chromosome end-to-end fusions detected in the absence of telomerase in Arabidopsis plants [35]. To answer this question we generated double mutant *Atercc1*/*Attert* and *Atrad1*/*Attert* Arabidopsis lines and compared their phenotypes with those of single *Atercc1*, *Atrad1* and *Attert* mutant lines in successive generations.

**Figure 1:**
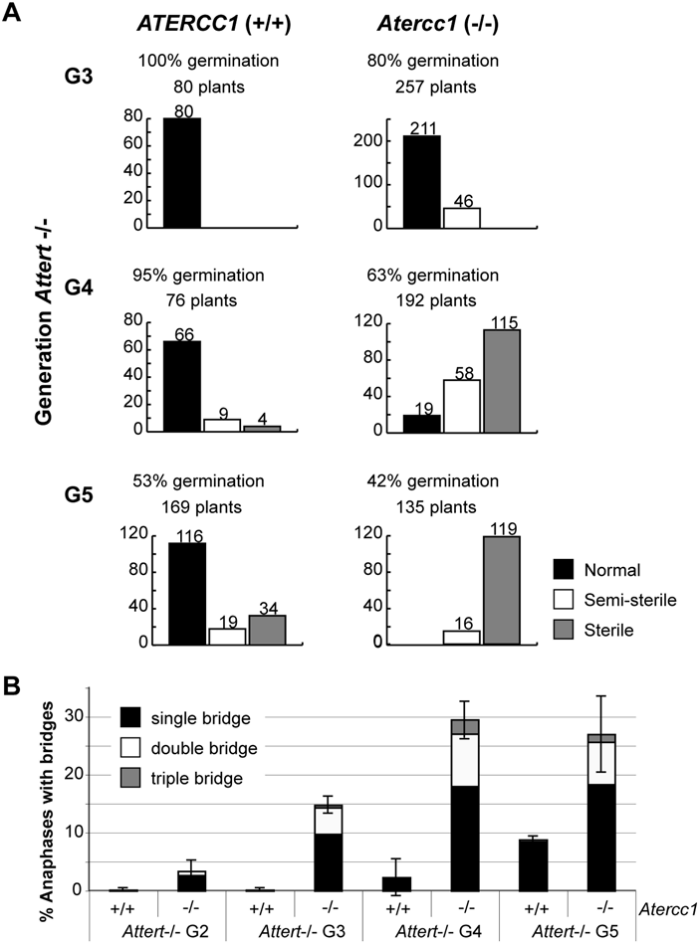
Accelerated genomic instability of *Attert/Atercc1* double mutants. A: Proportions of normal (black fill), semi-sterile (white fill) and sterile (grey fill) *Attert* (left) and *Attert/Atercc1* (right) mutants of generations G3, G4 and G5. With successive generations, increasing proportions of *Attert* mutant plants have reduced fertility or are sterile. This phenotype is considerably worsened in double *Attert/Atercc1* mutants. Single *Atercc1* and *Atrad1* mutants are fully fertile and develop normally (not shown). Percentage seed germination and numbers of plants counted in each class are given above the bars. B: Percentage of mitotic anaphases with one (black fill) or more (grey and white fills) chromosome bridges in *Attert* and *Atercc1/Attert* mutants, through *Attert* mutant generations G2, G3, G4 and G5. For each mutant and generation, 200–300 mitotic anaphases were examined from pistil cells. Error bars are +/− one standard deviation, from three independent experiments.

Homozygous *Attert* mutant plants were crossed to homozygous *Atercc1* and to *Atrad1* plants, to produce the doubly heterozygous F1 lines: *Attert/AtTERT Atercc1/AtERCC1* and *Attert/AtTERT Atrad1/AtRAD1*. Wild type, homozygous single *Attert*, *Atercc1*, *Atrad1* and double *Attert/Atercc1* and *Attert/Atrad1* F2 lines were selected and their growth and developmental phenotypes followed through successive generations of self-fertilisation. The original F2 lines are labelled Generation 1 (G1) for the *Attert* mutant status, and successive generations labelled G2, G3, …. At any given generation, plants were identified as belonging to one of three arbitrary phenotypic classes: wild-type (normal), semi-sterile (reduced fertility) or sterile (this class includes plants arrested in vegetative growth and those unable to produce viable seeds) (Figure S1). Single mutant *Atercc1* and *Atrad1* plants show wild-type phenotype and this is maintained over successive generations. *Attert* mutant plants show the expected progressive increase in both the proportion of plants presenting developmental defects and an increasing severity of these phenotypes over successive generations. The appearance and severity of these *Attert* phenotypes were however considerably advanced in the double *Atercc1/Attert* and *Atrad1/Attert* mutants. The results are presented in Figure 1A for the third, fourth and fifth (G3, G4, G5) telomerase mutant generation plants (see also Table S1). G3 *Atercc1/Attert* seeds show 80% germination efficiency, compared to 100% in G3 *Attert* single mutant plants. More importantly, 17.9% of *Atercc1/Attert* plants were semi-sterile while no obvious defects were visible in the single *Attert* mutant plants. By generation five (G5), no normal *Atercc1/Attert* plants were observed from a total of 135 plants, while 68% of the G5 *Attert* mutant plants were phenotypically normal. Equivalent results were obtained for the *Atrad1/Attert* double mutant (Table S1). Thus, absence of the nuclease AtERCC1/AtRAD1 induces a substantial acceleration of the *Attert*-associated developmental phenotype in Arabidopsis plants.

These observations raise the question of whether the accelerated developmental anomalies in *Atercc1/Attert* and *Atrad1/Attert* correlate with increased levels of cytogenetic damage in the double mutants. Successive generations of *Attert* mutant plants show progressive shortening of telomeres that eventually become uncapped and as a result end-to-end chromosomal fusions are generated. These fused, dicentric chromosomes can be detected as chromosome bridges at mitotic anaphase. We thus analyzed the frequencies of mitotic anaphase bridges in successive generations of the double and single mutant plants. For each mutant and generation, 200–300 mitotic anaphases were examined from pistil cells isolated from 3 individual plants (Table S2). As expected from their wild-type phenotype, no mitotic anaphases presenting bridges were detected in *Atercc1*, nor in *Atrad1* single mutant plants. Figure 1B presents the results for generations two to five (G2–G5) of double *Atercc1/Attert* and single *Attert* mutant plants. No anaphase bridges were observed in cells from the three first generations of single *Attert* mutant plants. In contrast, *Atercc1/Attert* double mutant plants show 4–5% of anaphases with chromosome bridges in generation two and 15% in generation three. By generation four, 30% of the anaphases prepared from *Atercc1/Attert* plants show chromosome bridges, compared to only 2–5% in the single *Attert* mutant third generation plants. Moreover, a higher proportion of anaphases presenting 2 or 3 bridges were observed in double mutant plants compared to the *Attert* single mutant plants. Equivalent results were obtained in anaphase preparations from pistil cells from *Atrad1/Attert* double mutant plants in which chromosome bridges appear 3 generations earlier compared to the *Attert* single mutant lines derived from the same cross (Table S2). Thus the accelerated *Attert* phenotype observed in *Attert* plants lacking the AtERCC1/AtRAD1 nuclease is directly correlated with an earlier onset of genomic instability in these plants. These results strongly suggest a protective role of AtERCC1/AtRAD1 proteins at short telomeres generated in the absence of telomerase. This effect of the AtERCC1/AtRAD1 proteins contrasts with that observed in mammalian cells with uncapped telomeres due to lack of TRF2, where the nuclease is essential for telomere fusion [15].

### Loss of AtERCC1/AtRAD1 Proteins Generates Extrachromosomal DNA

**Figure 2:**
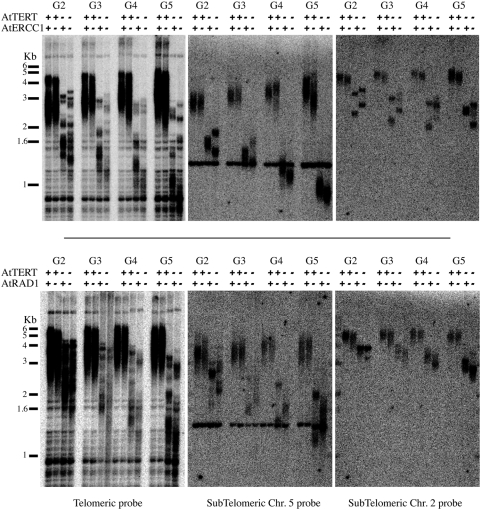
Telomere length measurements in *Attert*, *Atercc1*, *Atrad1* and double mutants. TRF analysis of bulk telomere lengths in DNA from flower buds of *Attert* mutant generations G2 to G5. Southern blots of Mbo1-digested total DNA from wild-type, *Attert*, *Atercc1*, and double *Atercc1*/*Attert* mutants (upper) and wild-type, *Attert*, *Atrad1*, and double *Atrad1*/*Attert* mutants (lower). Southern analysis using the telomeric repeat probe (left panels), and subtelomeric probes to chromosome 5 (middle panels) and chromosome 2 (right panels). Positions of DNA size markers are shown to the left. Wild-type controls are sister plants from the same original cross.

The simplest hypothesis to explain the accelerated appearance of genome instability is an increased rate of telomere erosion in the *Atercc1/Attert* double mutant plants. To test this hypothesis we carried out TRF analysis on DNA prepared from generations 2 to 5 of wild-type, *Atercc1*, *Atrad1* and *Attert* single mutants, and *Atercc1/Attert* and *Atrad1/Attert* double mutant plants (Figure 2). As expected from the absence of phenotype, telomeres of the *Atercc1* and *Atrad1* single mutant plants were maintained at the wild-type length through the four generations analyzed. A slight acceleration of telomere loss is observed in *Atercc1/Attert* and *Atrad1/Attert* double mutant plants, as compared to single *Attert* mutant plants. However, this cannot explain the appearance of fusions two generations earlier in double mutant plants. As shown in Figure 2, telomeres are longer in G2 *Atercc1/Attert* plants than in G4 *Attert* mutant plants, although the former have a greater proportion of mitoses with bridges (3.4%) than the latter (2.3%). Specific TRF analysis for the telomeres of the long arm of chromosome 2 and the short arm of chromosome 5, confirmed that telomeres in *Atercc1/Attert* G2 are longer that in G4 *Attert* in contrast with the similar number of anaphases with bridges detected in these plants (Figure 2). Similar results were obtained in later generations and in *Attert* plants lacking the AtRAD1 protein (Figure 2 and Table S2). Thus, increased telomere erosion in the absence of the AtERCC1/AtRAD1 endonuclease Arabidopsis plants cannot explain the acceleration of telomere dysfunction in the *Attert/Atercc1* and *Attert/Atrad1* plants.

**Figure 3:**
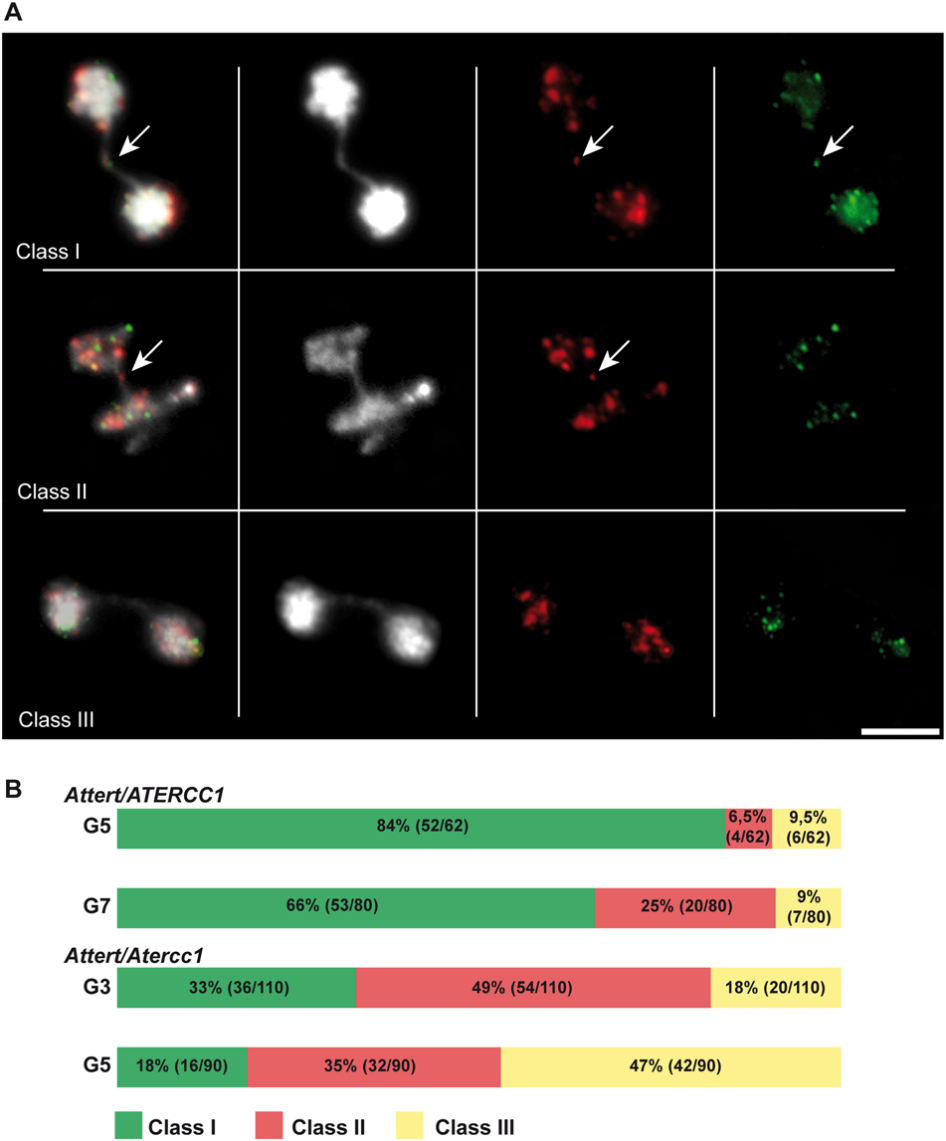
Fluorescence *in situ* hybridisation analysis (FISH) of the structure and origin of dicentric chromosome bridges. A: Examples of FISH analysis of mitoses from flower pistils: from right to left, fluorescent probes to telomeric repeat DNA (green), pooled 10 subtelomeric BACs (red), DAPI-stained DNA (white) and merged image. From top to bottom: examples of anaphase bridges of class I (both subtelomeric and telomeric foci in bridge), class II (only subtelomeric foci present in the bridge) and class III (bridges with neither subtelomeric nor telomeric foci). The scale bar represents 5 µm. B: Distribution of the three classes of anaphase bridges in *Attert* (generations G5, G7) and *Atercc1/Attert* (G3, G5) mutant plants. Relative percentages and numbers counted in each class, mutant and generation are indicated in the bars.

The alternative hypothesis is that the AtERCC1/AtRAD1 proteins protect short telomeres against recombination. We thus carried out fluorescence *in situ* hybridisation (FISH) analyses of chromosome fusions using telomeric-repeat and subtelomeric probes (a pool of BACs corresponding to the two ends of each of the five Arabidopsis chromosomes). The subtelomeric BAC FISH probes have been previously validated by Fibre-FISH [36]. Three categories of anaphase bridges could be detected in the FISH analyses, those corresponding to end-to-end chromosome fusions presenting subtelomeric signals with (class I) or without (class II) telomeric repeats, and bridges lacking both subtelomeric and telomeric signals (class III) (Figure 2A). The proportions of the three classes of anaphase bridges were determined in *Attert* plants at G5 (10% of anaphases with bridges) and G7 (25% anaphases with bridges). As expected, in correlation with the increased loss of telomere repeats in G7 plants, the proportion of bridges lacking telomeric signals is increased with respect to G5 plants (25% versus 6,5%). This increase was accompanied by a corresponding reduction in the proportion of bridges with telomeric repeats (G5 84%, G7 66%). No changes were seen in the proportion of class III bridges lacking both subtelomeric and telomeric signals (G5 9,5%, G7 9%) (Figure 3B). In contrast a substantial increase in the proportions of class II and III bridges was observed in *Atercc1/Attert* mitoses. Thus, G3 *Atercc1/Attert* cells that present a similar proportion of anaphases with bridges to G5 *Attert* cells (10–15%), show 49% of class II (versus 6,5% in *Attert* cells) and 18% of class III (versus 9% in *Attert* cells) (Figure 3B). Strikingly, the proportion of class III bridges increases up to 47% in *Atercc1/Attert* G5 cells, versus 9% in G7 *Attert* cells which show the same proportion of anaphases with bridges (25%). Thus, by G5 almost half of the anaphase bridges in *Atercc1/Attert* cells do not result from chromosome end-to-end fusions. Similar results were obtained for *Atrad1/Attert* cells (data not shown). These observations suggest strongly that AtERCC1/AtRAD1 endonuclease protects short telomeres against other types of recombination than the fusion of uncapped chromosomes ends.

**Figure 4:**
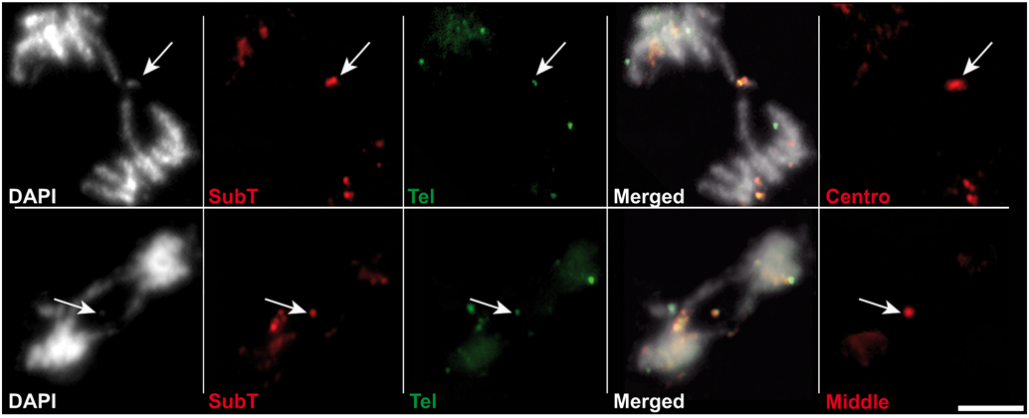
Identification of extrachromosomal DNA containing subtelomeric and telomeric signals. 90% of *Attert/Atercc1* class III mitotic figures (anaphase bridges with neither subtelomeric nor telomeric foci) present an acentric chromosome fragment (arrows) that hybridises with both subtelomeric and telomeric probes. Rehybridisation of these slides with pools of 10 BAC probes located in the middle of each of the ten Arabidopsis chromosome arms (Middle) or near to the centromere (Centro). All extrachromosomal DNA fragments hybridized with both probe sets indicating that they consist of (at least the major part) whole chromosome arms. Two mitotic figures (upper, lower) with DAPI-stained DNA (white), sub-telomeric (red), telomeric (green) and merged images are shown from left to right. Rightmost images show rehybridisation with centromere-proximal (upper) and middle-arm (lower) probe sets (red). The scale bar represents 5 µm.

Careful observation of the FISH images of mitotic *Atercc1/Attert* cells revealed extrachromosomal DNA masses in 90% of the anaphases presenting class III bridges (Figure 4). In all cases this extrachromosomal DNA hybridized both with subtelomeric and telomeric repeat FISH probes. To better understand the nature of these extrachromosomal DNAs, we realized FISH analyses using a pool of 10 BAC probes situated in the middle of each arm of the five Arabidopsis chromosomes. All the analyzed anaphase figures containing extrachromosomal DNA produced a positive signal. Identical results were obtained with a mix of centromere-proximal BAC probes specific for each chromosome arm. Equivalent results were obtained for *Atrad1/Attert* double mutant plants (data not shown). Class III bridges in *Atercc1/Attert* and *Atrad1/Attert* are thus associated with the generation of acentric DNA corresponding to a chromosome arm in at least 90% of cases.

### AtERCC1/AtRAD1 Proteins Protect Shortened Telomeres from the Action of Recombination

**Figure 5:**
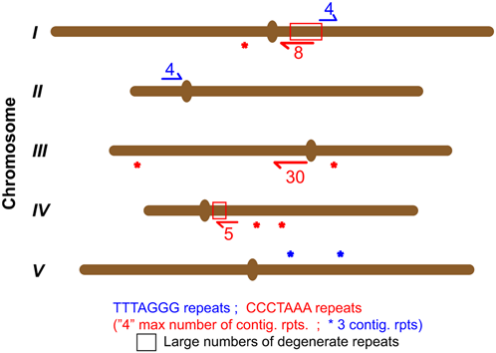
Interstitial telomeric repeat in Arabidopsis thaliana genome. (TTTAGGG)_n_ (blue) and (CCCTAAA)_n_ (red) interstitial telomeric repeat loci shown on the five Arabidopsis chromosomes. The number of perfect repeats (numbers or asterisk for triple-repeats) is given for each locus. Two extensive regions of degenerate (CCCTAAA)_n_ repeats were identified on chromosome 1R (349 Kb) and 4R (67 Kb). Centromere positions are indicated as bulges in the chromosomes. Details are given in the text.

In order to explain these data, we considered the hypothesis that in the absence of AtERCC1/AtRAD1, short telomeres unable to form a T-loop could invade internal telomere-related sequences, in a similar manner to the events proposed to generate telomeric double minute chromosomes in ERCC1-deficient mouse cells [15]. With only 5 chromosome pairs and a fully sequenced genome, Arabidopsis is a particularly good model for the dissection of such events. We thus used the sequence viewer of the Arabidopsis Information Resource (TAIR) (http://www.arabidopsis.org/servlets/sv) and the “fuzznuc” program of the EMBOSS suite [37] to map and characterise interstitial telomeric repeats in the Arabidopsis genome, presented in schematic form in Figure 5. The bioinformatics search of sequences revealed the presence of 4 contiguous perfect TTTAGGG repeats on chromosomes 1R and 2L and three repeats at two loci on chromosome 5R. Searching for the CCCTAAA sequence identified three loci with 8, 30 and 5 contiguous perfect repeats on chromosome arms 1R, 3L and 4R respectively. Five loci of three contiguous repeats are found on chromosomes 1L, 3L, 3R and 4R. Of particular interest are two extensive regions of degenerate telomeric repeats identified on chromosome 1R (349 Kb) and 4R (67 Kb). 5.9% of the chromosome 1R region DNA consists of perfect C-strand telomere repeats (CCCTAAA)_n_, a figure which rises to 17.2% if repeats with 1 mismatch are included. G-strand repeats (TTTAGGG)_n_ are considerably less represented in this block, with 0.05% and 1.3% respectively. Similarly the chromosome 4R region has 8.8% (perfect) and 25.18% (including 1 mismatch) C-strand repeats and 0 (perfect) and 0.8% (1 mismatch) G-strand repeats. The longest perfect tandem C-strand repeats in these regions are an 8-mer in the 1R region and a 5-mer in the 4R region. On the G-strand, the longest perfect tandem repeat in these regions is a 3-mer in the 1R region, with none in the 4R region.

**Figure 6:**
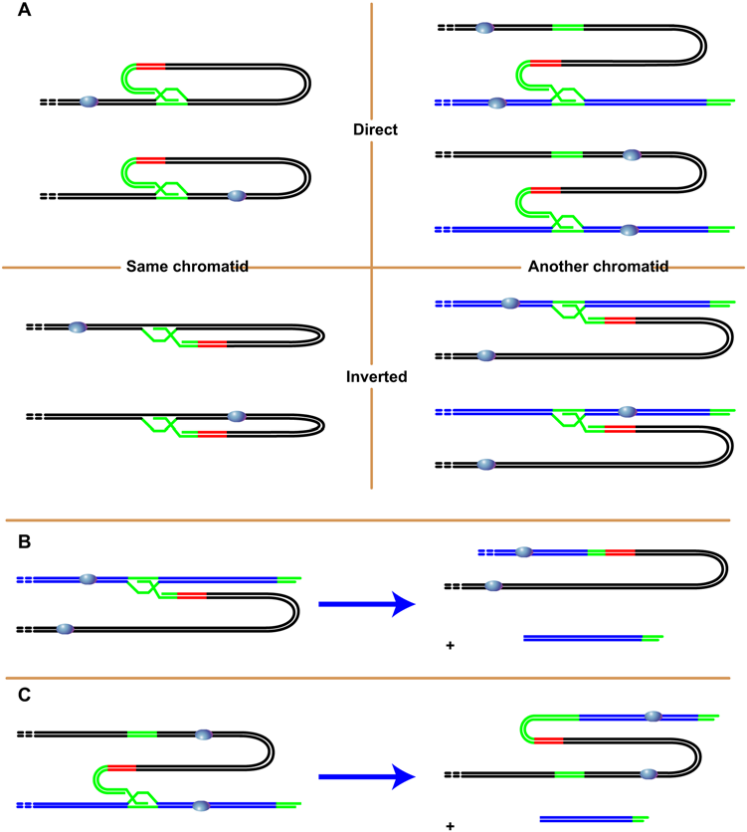
Possible interstitial invasion configurations and outcomes. Invasion of the same (left) or another (right) chromatid in direct (top) or inverted (bottom) orientations gives eight possible configurations of telomere invasion of interstitial telomeric-repeat DNA. (A) Resolution of only two of these eight configurations will result in the co-incident production of a dicentric and an acentric chromosome (B,C). Telomeric-repeat DNA (green), subtelomeric regions (red) and centromeres (balls) are indicated.

Should a telomeric 3′-ended G-overhang recombine and crossover with interstitial telomeric repeat sequences, the consequences would depend upon the orientation of these sequences relative to the centromere(s) and whether or not they are on the same chromatid or chromosome arm. In all, there are eight possible invasion configurations (Figure 6A). Holliday junction resolution of only two of these would generate the observed class III dicentric chromosome (anaphase bridge lacking subtelomeric and telomeric repeats) and an acentric chromosome, as illustrated in Figure 6B and 6C. Figure 6B shows invasion by another chromosome (or chromatid) of (CCCTAAA)_n_ sequences, such as those present in the extensive blocks on the right arms of chromosomes 1R and 4R. Figure 6C shows invasion by another chromosome (or chromatid) of the (TTTAGGG)_n_ sequence, such as that present on the left arm of chromosome 2. In both cases resolution of the resulting Holliday junction can give rise to a dicentric and an acentric chromosome.

**Figure 7:**
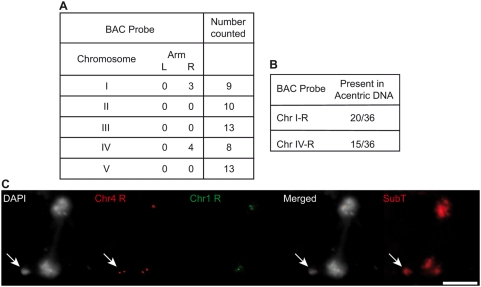
Acentric DNA fragments are the right arms of chromosomes 1 and 4. (A) *Attert/Atercc1* mitotic figures with acentric DNA were hybridized with individual BAC probes specific to the right and left arms of each of the five chromosomes in independent experiments. Only the probes to the right arms of chromosomes 1 and 4 hybridized to acentric fragments. Numbers of mitotic figures analysed are given to the right for each probe pair. (B) Repeating this analysis with only the probes for the right arms of chromosomes 1 and 4. Panel (C) shows an example of the FISH analysis with, from left to right: DAPI-stained DNA (white), BAC probes to the right arm of chromosome 4 (red) and 1 (green) and the merged image. Rehybridisation with the pooled subtelomeric BAC probe set is shown in the rightmost image (SubT). Acentric fragments are arrowed. The scale bar represents 5 µm.

According to this model, the acentric DNA observed in *Atercc1/Attert* Arabidopsis cells should correspond to chromosome arms 1R, 4R and possibly, 2L. To test this prediction, we carried out FISH analyses using BAC probes corresponding to all 10 individual Arabidopsis chromosome arms. The results in Figure 7A show that only probes for the right arms of chromosomes 1 and 4 hybridized with the extrachromosomal DNA. We confirmed these results with FISH using only the probes to Chr. 1R (red) and Chr. 4R (green) – the extrachromosomal DNA in 35 out of the 36 anaphases examined hybridised to one of these two probes (Figure 7B,C). The acentric fragments thus correspond to the chromosome arms distal to the two extensive blocks of interstitial telomeric DNA and these data thus strongly support the origin of the observed dicentric+acentric chromosomes in *Atercc1/Attert* plants through homologous recombination of telomeric and interstitial telomere-repeat DNA sequences.

## Discussion

We present here an analysis of the roles of the structure-specific ERCC1/XPF (AtERCC1/AtRAD1) endonuclease in telomere homeostasis in the plant *Arabidopsis thaliana*. Double *Atercc1/Attert* or *Atrad1/Attert* mutant Arabidopsis plants show considerably more severe growth and developmental phenotypes than single *Attert* mutant plants. This aggravation of the telomerase mutant phenotype is directly correlated with an earlier onset of chromosome instability, as detected by the appearance of mitotic dicentric anaphase bridges. Analysis of the structure of these dicentric chromosomes shows that, in contrast to *Attert* plants where 90% of dicentrics result from end-to-end fusion, 50% of dicentrics in *Atercc1/Attert* cells result from recombination of telomeres with two extensive regions of interstitial telomere-related DNA in the Arabidopsis genome.

In mammalian cells the ERCC1/XPF heterodimer is associated to telomeres through interaction with TRF2 [15]. The rapid ageing phenotype of *ERCC1*−/− and *XPF*−/− mice has been attributed to roles of these proteins in DNA repair mechanisms other than NER, such as the repair of DNA interstrand cross-links (44,45) and DSB (46,47). Cytogenetic analyses of ERCC1−/− mouse embryonic fibroblasts (MEFs) show neither defects in telomeres nor in telomeric G-strand homeostasis and no end-to-end chromosomes fusions were detected in these cells. However, FISH analysis on metaphase spreads of ERCC1−/− MEF cells showed greatly elevated numbers of telomere-containing double-minute chromosomes (TDMs), compared to wild-type and XPC−/− controls [15]. This generation of double minute chromosomes presumably contributes to the severe postnatal growth defects and death at 3 weeks of mice mutated for either protein [38]–[42].

Although the roles of the ERCC1/XPF nuclease in NER and double strand break repair are conserved in Arabidopsis [17],[18],[21], Arabidopsis *Atercc1* and *Atrad1* mutants grow and develop normally and show no detectable chromosomal instability, with neither anaphase bridging nor alterations in bulk telomere length detected in these plants after more than five mutant generations (this work). The situation is however strikingly different in plants also lacking telomerase, in which absence of either the AtERCC1 or AtRAD1 proteins dramatically advances the appearance of developmental defects and chromosomal instability.

As with many other organisms including mammals, absence of telomerase leads to progressive shortening of telomeric repeat arrays, destabilising the T-loop structure at telomeres and resulting in their recognition by the cellular recombination machinery. Recombination of these shortened, non-functional telomeres principally results in end-to-end chromosomal fusions and dicentric chromosomes (reviewed by [10]). However, overhanging G-strand telomeric DNA from non-functional telomeres could also invade and recombine with interstitial telomere-like sequences. Such recombination between telomeres and interstitial sequences would have differing consequences, depending on the location and orientation of these interstitial sequences with respect to the centromere. Studies in cultured human cells suggest that the ERCC1/XPF endonuclease would play two roles in the avoidance of such events: removal of G-strand overhangs at decapped telomeres would reduce the propensity of these to invade cognate interstitial sequences and should such invasion occur, ERCC1/XPF cleavage of the intermediate structure would pre-empt resolution by the recombination machinery [15].

In this work we report strong developmental phenotypes in *Atercc1/Attert* (and *Attert/Atrad1*) Arabidopsis plants, associated with the frequent occurrence of mitoses with dicentric and acentric chromosomes. Southern analysis shows little or no acceleration of bulk telomere shortening in the double *Atercc1/Attert* and *Attert/Atrad1* mutants, compared to single *Attert* plants. Thus, although a minor contribution of the absence of AtERCC1/AtRAD1 to telomere shortening in *Attert* mutants cannot be ruled out, this cannot explain the dramatic acceleration of the developmental and chromosomal instability phenotypes observed in the double mutants. This conclusion is reinforced by the striking decrease in the relative proportion of end-to-end (Class I) chromosomal fusions in double *Atercc1/Attert* and *Attert/Atrad1* mutants compared to single *Attert* mutants. This is more than compensated by relative increases in proportions of mitoses with dicentric bridges lacking telomeric (Class II) or both telomeric and sub-telomeric (Class III) DNA in double mutants. Furthermore, acentric chromosome arms are observed in 90% of mitoses with Class III dicentric bridges in the double mutants. Absence of AtERCC1/AtRAD1 thus both strongly increases the numbers and affects the nature of chromosomal fusions in *Attert* mutants.

**Figure 8:**
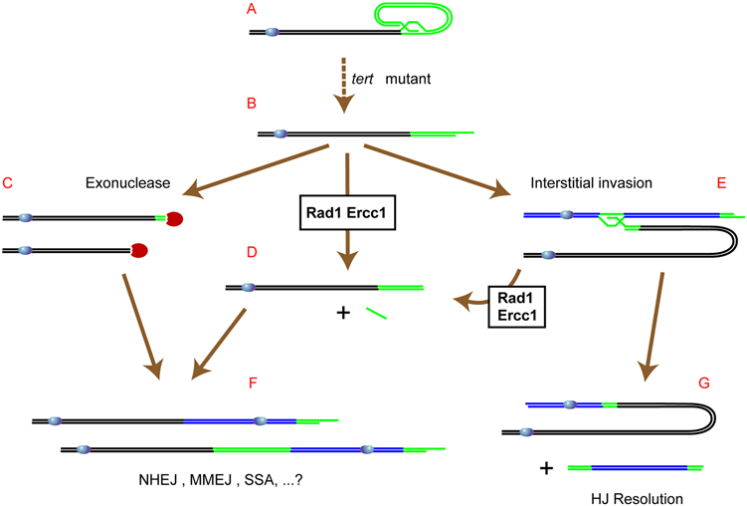
Model of the roles of AtERCC1/AtRAD1 in different fates of uncapped telomeres in *Attert* mutant Arabidopsis. Erosion of telomeric repeat DNA in the *Attert* mutant leads to progressively more frequent loss of T-loop structure and uncapping of telomeres (A–>B). Uncapped telomeres may be further eroded by exonucleases (B–>C) or AtERCC1/AtRAD1 can cleave the G-strand overhang to leave a blunt end (B–>D). Non-homologous, micro-homology mediated, or single-strand annealing recombination (NHEJ, MMJ, SSA) can fuse chromosomes of these structures (C–>F and D–>F). The G-strand overhang of structure (B) can also recombine with interstitial telomeric repeat sequences (E) and in certain invasion configurations (Chr 1R and 4R in Arabidopsis), resolution of this structure by Holliday-junction resolvase generates a dicentric plus an acentric chromosome (G). Cleavage of structure (E) by AtERCC1/AtRAD1 prior to the action of resolvase will produce structure (D), although the existence of this AtERCC1/AtRAD1-dependent process (E–>D) cannot be verified in Arabidopsis, given that evidence for structure (E) is only found in the absence of AtERCC1/AtRAD1.

In order to determine whether these dicentric+acentric figures could result from recombination between telomeres and interstitial telomeric-sequences, we analysed the numbers and positions of internal telomere-related sequences in the fully sequenced Arabidopsis genome. This bioinformatics analysis shows the presence of 2 extensive blocks of degenerate telomere sequence on the right arms of Arabidopsis chromosomes 1 and 4. Invasion of these interstitial telomere-related sequences by the G-strand of a decapped telomere would create a recombination intermediate, processing of which by the homologous recombination machinery could generate the observed dicentric+acentric mitotic figures (Figure 8: B–>E–>G). Our analysis predicts that the acentric chromosomes should correspond to the right arms of either chromosome 1 or 4, a prediction confirmed in 35/36 of such acentrics examined.

The processes leading to the events which we describe in Arabidopsis plants (dicentric chromosome+acentric arm; Figure 8G) thus appear equivalent to those resulting in the TDMs in human cells described by Zhu *et al* [15]. The striking difference between our data and that in animal cells is the normal growth and development and the absence of karyotypic abnormalities in (*AtTERT*+) *Atercc1* or *Atrad1* mutant plants. The karyotypic instability of *Atrad1* and *Atercc1* mutants thus depends upon the absence of telomerase. This implies that interstitial telomere invasions do not occur, or are very rare in wild-type plants, in contrast to the observations in cultured human cells, where such invasion events are presumably very frequent (TDMs observed in 44–86% of mitoses in different ERCC1−/− cell lines).

It is striking to note the different outcomes of equivalent recombination processes in the different organisms. Homologous recombination of telomeres with interstitial sequences in direct orientation on the same chromatid arm is proposed to generate massive chromosome breakage and circular acentrics (TDMs) in mouse cell culture, while recombination of telomeres with interstitial sequences in inverted orientation on another chromatid arm leads to breakage and fusion of two specific chromosome arms in Arabidopsis. These differences thus appear to primarily depend upon the structure of the genome/karyotype and the locations of the interacting sequences. This striking illustration of the different outcomes and impacts of recombination processes in the different genomic contexts is underlined by recent data from the fission yeast, *S. pombe* [43].

Absence of telomerase (Trt1) in *S. pombe* leads to telomere shortening and cell death. However, rare “survivor” cells escape and grow normally due to circularisation of their chromosomes. A recent report shows that the absence of Rad16 has no effect on the rate of telomere shortening in *trt1* cells, but strongly reduced the occurrence of survivors (Rad16 is the *S. pombe* XPF ortholog). In a series of elegant experiments, these authors further show that the chromosome circularisation leading to survival of *trt1* cells occurs through Rad16-dependent, single-strand annealing (SSA) recombination between homology regions present as inverted repetitions between 7 and 13 Kb from the telomeres of chromosomes I and II [43]. As in Arabidopsis and animal cells, absence of *S. pombe* Rad16 protein thus profoundly affects the recombination of de-protected telomeres, at least in this selected subset of “survivor” events. In Arabidopsis *Atercc1/Attert* plants, end-to-end chromosome fusions represent 53% of (total) anaphase bridges, which thus cannot have been generated through the ERCC1/XPF -dependent SSA recombination pathway. We are currently initiating work to elucidate the roles of the different homologous and non-homologous recombination pathways in the generation of these fusions.

## Materials and Methods

### Arabidopsis Mutants and TRF Analysis

*Arabidopsis thaliana* plants were grown in soil in the greenhouse under standard conditions. The *Attert* [44], *Atercc1* [18] and *Atrad1* [45] Arabidopsis mutants have been described previously. The two double *Atrad1*/*Attert* and *Atercc1*/*Attert* mutants were produced by crossing *Atrad1* and *Atercc1* homozygotes with an *Attert* homozygote (3rd mutant generation), using standard techniques. PCR genotyping was carried out as described for *Attert* [44], *Atrad1* [19] and *Atercc1* [18]. TRF analysis of telomere length in Mbo1-digested genomic DNA was as previously described for the telomeric and subtelomeric chromosome 2 probes [46] and for subtelomeric 5 probe [47].

### DAPI Staining of Mitoses

Whole flower buds were collected and fixed, pistils were digested and were squashed on slides [48]. Slides were mounted using Vectashield (Vector Laboratories) mounting medium with 1.5 µg/ml DAPI (4′,6-Diamidino-2-Phenylindole) and observed by fluorescence microscopy, using a Zeiss Imager.Z1 microscope. Images were further processed and enhanced using Adobe Photoshop software.

### Fluorescence *In Situ* Hybridisation (FISH)

BACs from subtelomeric regions of Arabidopsis chromosomes (F6F3, F23A5, F15B18, F17A22, F4P13, T20O10, F6N15, T19P19, F7J8, K9I9), the middle of chromosome arms (F12K11, F20D21, T8K22, F12C20, K1G2, F16L2, T5K18,T1A4, MIJC20) [49] and centromere-proximal regions (F12K21, F2J6, T25N22, T10F5, T4A2, T5C2, T32N4, T32A17, T8M17, F5H8) [49] were labelled with biotin-16-dUTP or digoxigenin-11-dUTP (Roche) using the BioPrime DNA labelling system (Invitrogen) and telomeric probe was labelled by PCR [(95°C 1′, 55°C 40″, 72°C 2′)×5 (94°C 1′, 60°C 40″, 72°C 2′)×25] with digoxigenin-11-dUTP using specific telomere primers 5′(TTTAGGG)_6_3′. FISH experiments were performed according to [50], as previously described [36]. For the detection of biotin-labelled probes, avidin∶Texas Red (1∶500, Vector Laboratories) followed by goat anti-avidin∶biotin (1∶100, Vector Laboratories) and avidin-Texas Red (1∶500) were used. Mouse anti-digoxygenin (1∶125, Roche) followed by rabbit anti-mouse∶fluorescein isothiocyanate (FITC) (1∶500, Sigma) and goat anti-rabbit∶Alexa 488 (1∶100, Molecular Probes) were used for the detection of digoxygenin-labelled probe. For multiple hybridisations of the same slide, FISH was carried out according to Mokros *et al* [51], using BACs labelled either with Cy5-dUTP or Cy3-dUTP (Amersham) by standard nick translation reactions (Roche).

## Supporting Information

Figure S1 Phenotypes of *Atercc1/Attert* plants. Photographs of normal, semi-sterile and sterile *Atercc1/Attert* mutants.
(10.70 MB TIF)

Table S1 Developmental defects in *Attert* versus *Attert/Atrad1* and *Attert/Atercc1* mutants.
(0.05 MB DOC)

Table S2 Quantification of mitoses with one or more anaphase bridges in *Attert* versus *Attert/Atrad1* and *Attert/Atercc1* mutants.
(0.05 MB DOC)
