# Supplementary figures and images for: ERCC1/XPF Protects Short Telomeres from Homologous Recombination in Arabidopsis thaliana
Source: PLoS Genet. 2009 Feb 13;5(2):e1000380. doi: 10.1371/journal.pgen.1000380 (PMC2632759; doi:10.1371/journal.pgen.1000380)

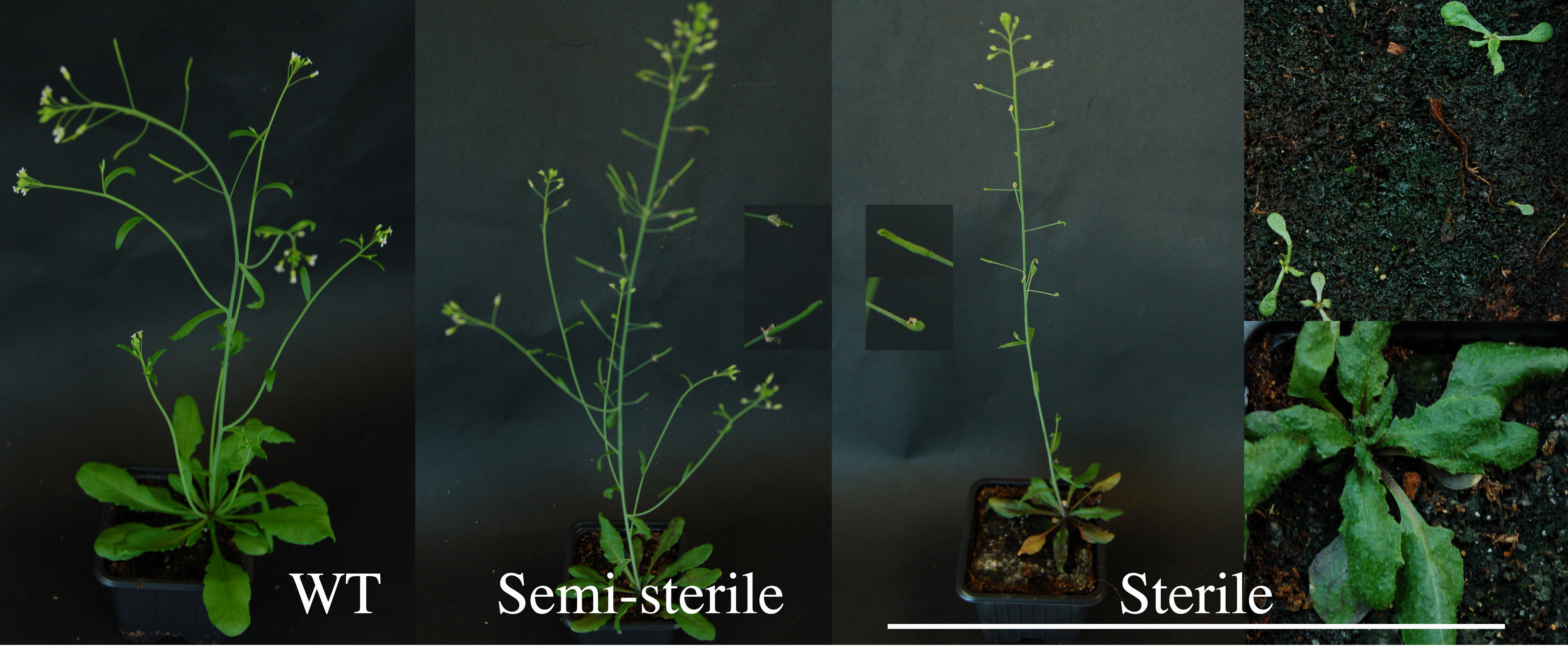

Supplement: Figure S1 — Phenotypes of Atercc1/Attert plants. Photographs of normal, semi-sterile and sterile Atercc1/Attert mutants. (10.70 MB TIF) [file pgen.1000380.s001.tif]
