# Supplementary material for: ERCC1/XPF Protects Short Telomeres from Homologous Recombination in Arabidopsis thaliana
Source: PLoS Genet. 2009 Feb 13;5(2):e1000380. doi: 10.1371/journal.pgen.1000380 (PMC2632759; doi:10.1371/journal.pgen.1000380)
Supplement: Table S1 — Developmental defects in Attert versus Attert/Atrad1 and Attert/Atercc1 mutants. (0.05 MB DOC) [file pgen.1000380.s002.doc]

**Table S1.**

Developmental defects in *Attert* versus *Attert/Atrad1* and *Attert/Atercc1* mutants.

|  | Total seeds | Germinated  (percentage) | Normal | Semi-sterile | Sterile |
| --- | --- | --- | --- | --- | --- |
| ***AtERCC1*** | | | | | |
| *Attert G2* | 80 | 80 (100) | 80 | 0 | 0 |
| *G3* | 80 | 76 (95) | 66 | 9 | 4 |
| *G4* | 320 | 169 (53) | 116 | 19 | 34 |
| *G5* | 320 | 205 (68) | 99 | 25 | 81 |
| ***Atercc1*** | | | | | |
| *Attert G2* | 320 | 257 (80) | 211 | 46 | 0 |
| *G3* | 304 | 192 (63) | 19 | 58 | 115 |
| *G4* | 320 | 135 (42) | 0 | 16 | 119 |
| *G5* | 320 | 109 (35) | 0 | 17 | 92 |
| ***AtRAD1*** | | | | | |
| *Attert G2* | 160 | 150 (94) | 144 | 0 | 6 |
| *G3* | 160 | 134 (84) | 115 | 10 | 9 |
| *G4* | 160 | 85 (54) | 49 | 20 | 16 |
| *G5* | 320 | 223 (70) | 93 | 44 | 86 |
| ***Atrad1*** | | | | | |
| *Attert G2* | 320 | 281 (88) | 272 | 6 | 1 |
| *G3* | 320 | 234 (73) | 151 | 36 | 47 |
| *G4* | 320 | 121 (38) | 10 | 48 | 63 |
| *G5* | 320 | 104 (32) | 0 | 8 | 96 |
