# Supplementary material for: ERCC1/XPF Protects Short Telomeres from Homologous Recombination in Arabidopsis thaliana
Source: PLoS Genet. 2009 Feb 13;5(2):e1000380. doi: 10.1371/journal.pgen.1000380 (PMC2632759; doi:10.1371/journal.pgen.1000380)
Supplement: Table S2 — Quantification of mitoses with one or more anaphase bridges in Attert versus Attert/Atrad1 and Attert/Atercc1 mutants. (0.05 MB DOC) [file pgen.1000380.s003.doc]

**Table S2.**

Quantification of mitoses with one or more anaphase bridges in *Attert* versus *Attert/Atrad1* and *Attert/Atercc1* mutants.

|  | Anaphases scored | Bridges | Mean (std. dev.) |
| --- | --- | --- | --- |
| ***AtERCC1*** | | | |
| *Attert G2* | 600 | 1 | 0.17 (0) |
| *G3* | 600 | 1 | 0.17 (0) |
| *G4* | 600 | 16 | 2.33 (0) |
| *G5* | 600 | 53 | 8.83 (0) |
| ***Atercc1*** | | | |
| *Attert G2* | 547 | 19 | 3.4 (0) |
| *G3* | 569 | 167 | 14.8 (3) |
| *G4* | 600 | 104 | 29.53 (8.5) |
| *G5* | 500 | 141 | 27 (5) |
| ***AtRAD1*** | | | |
| *Attert G2* | 600 | 0 | 0 (0) |
| *G3* | 600 | 0 | 0 (0) |
| *G4* | 600 | 14 | 2.33 (0) |
| *G5* | 864 | 72 | 7.86 (0) |
| ***Atrad1*** | | | |
| *Attert G2* | 360 | 9 | 2.37 (0) |
| *G3* | 400 | 42 | 11.17 (0) |
| *G4* | 600 | 71 | 10.83 (1) |
| *G5* | 843 | 120 | 16.4 (2) |
